# Semi-Supervised Radar Work Mode Recognition Based on Contrastive Learning

**DOI:** 10.3390/s25247440

**Published:** 2025-12-07

**Authors:** Peishan Sun, Mingyang Du, Zhihui Li, Xuan Chen, Junpeng Shi

**Affiliations:** College of Electronic Countermeasure, National University of Defense Technology, Hefei 230071, China; sunpeishan123@nudt.edu.cn (P.S.); lizhihui20@nudt.edu.cn (Z.L.); chenxuan24a@nudt.edu.cn (X.C.); shijunpeng20@nudt.edu.cn (J.S.)

**Keywords:** contrastive learning, mode recognition, semi-supervised learning, missing pulse, false pulse

## Abstract

**Highlights:**

**What are the main findings?**

- An end-to-end semi-supervised learning framework based on triple-branch contrastive learning is proposed for fine-grained radar work mode recognition in complex electromagnetic environments.
- Trained with only 10% labeled data, the framework outperforms state-of-the-art contrastive learning methods by 17% and 34% in recognition accuracy on PDW-MP and PDW-FP datasets, respectively, and maintains robustness in non-ideal scenarios.

**What is the implication of the main finding?**

- The high accuracy under limited labeled data significantly alleviates the reliance on costly and time-consuming radar data annotation, lowering the threshold for practical deployment of semi-supervised learning in radar systems.
- Robust performance in non-ideal scenarios validates the framework’s applicability to real-world complex electromagnetic environments, providing a reliable solution for fine-grained radar mode recognition tasks.

**Abstract:**

Deep learning for fine-grained radar mode recognition faces a major bottleneck—its heavy reliance on expensively labeled data. To address this, we propose a novel semi-supervised framework that effectively leverages unlabeled data. Through an end-to-end, triple-branch framework that integrates a dual contrastive learning mechanism with tailored strategies for pulse distortions, our model achieves high accuracy with minimal labels. Experimental results on two challenging datasets demonstrate that the proposed framework boosts accuracy by 17% to 34% using only 10% of the labeled data, establishing a new state-of-the-art performance.

## 1. Introduction

Modern Electronic Warfare (EW) primarily consists of three key components: Electronic Support (ES), Electronic Attack (EA), and Electronic Protection (EP) [1,2]. ES is responsible for searching, locating, and analyzing intercepted electronic signals to provide decision-making support for other systems. Modern Multifunction Radar (MFR) can leverage programmable parameters and flexible modulation types to generate fine-grained radar work modes, effectively meeting diverse mission requirements [3,4,5,6]. The recognition of MFR modes is critical for ES missions and plays an important role in the fields of electronic intelligence, cognitive radio, and operational decision-making [7,8,9]. The reliable assessment of behavioral posture and potential threats posed by non-cooperative MFRs is a vital and challenging task for ES systems. The key to effective identification lies in learning and distinguishing the patterns presented by the characteristic pulse parameters and waveforms of MFR.

MFRs can flexibly regulate modulation types and parameters to generate diverse fine-grained work modes, enhancing mission adaptability and anti-detection capability [10]. These modes are characterized by pulse descriptor word (PDW) parameters, including frequency (RF), pulse width (PW), and pulse repetition interval (PRI), with each parameter combination representing a distinct pattern. Identifying these modes through inter-pulse parameter analysis provides critical intelligence for electronic surveillance. Recent deep learning advances have demonstrated superior performance in MFR signal analysis. He et al. [11] embedded functional semantics into radar waveforms using LSTM networks, shifting focus from signal properties to behavioral understanding. To address noisy environments, Zhang et al. [12] developed a Residual Shrinkage Convolutional Network that suppresses irrelevant features through adaptive thresholding. Xiong et al. [13] integrated U-Net denoising with multi-scale attention for robust pattern recognition. For uncertainty modeling, Bayesian approaches have shown promise. Du et al. [14] proposed a Bayesian Attention Belief Network using CNN priors to guide inference, while He et al. [15] employed Sparse Bayesian Learning for joint parameter estimation in multipath environments. These methods enhance reliability under dynamic signal conditions. Supervised radar classification faces practical constraints due to the high cost of manual pulse annotation and the difficulty of obtaining quality labels in complex electromagnetic environments. While semi-supervised learning, leveraging both labeled and unlabeled data, offers a promising alternative, existing methods remain susceptible to pulse distortion under non-ideal conditions with limited labeled samples, requiring improved recognition performance.

Under non-ideal conditions with limited labeled samples, the learned feature distributions of existing semi-supervised recognition methods are susceptible to pulse distortion. Therefore, the recognition performance under non-ideal conditions with a small number of labeled samples still needs to be improved. This paper proposes a Tri-branch Semi-supervised Contrastive Learning (TriSCL) approach for radar work mode recognition based on a semi-supervised framework. This paper makes the following key contributions to advance fine-grained radar work mode recognition under limited labeled data and non-ideal conditions:

1. We design a novel three-branch contrastive learning framework. Distinctively, the third branch receives a fusion of the augmented views from the first two branches, serving as a stable anchor. This allows the model to compute more consistent unsupervised contrastive losses, which are then jointly optimized with supervised objectives to effectively tackle label scarcity.
2. We enhance model robustness against realistic signal degradation by designing two dedicated augmentation strategies that simulate false pulses and pulse loss. Integrated into the framework, they force the model to learn distortion-invariant representations.
3. We introduce an encoder equipped with residual shrinkage modules and dual attention to improve feature extraction. This architecture selectively focuses on critical temporal patterns and feature dependencies while suppressing noise, which is crucial for handling complex radar modulations.
4. We conduct Extensive experiments on the PDW-MP and PDW-FP datasets, demonstrating state-of-the-art performance. Our method achieves an average accuracy improvement of 15% using only 10% labeled data, proving its efficacy and practical value.

The structure of this paper is organized as follows. The subsequent section (Section 2) commences with a review of related work in radar emitter recognition and contrastive learning. Section 3 then recalls the foundational concepts of radar pulse stream structure and diverse inter-pulse modulation types. The core of our work—TriSCL—is thoroughly designed and presented in Section 4. Section 5 is dedicated to an extensive experimental evaluation, where the accuracy and robustness of our method are assessed against several state-of-the-art techniques. The paper concludes with a summary of findings and a perspective on future work in Section 6.

## 2. Related Work

### 2.1. Semi-Supervised Radar Emitter Identification Methods

Several semi-supervised learning frameworks have been adapted for the radar emitter identification, which can be categorized as follows:

#### 2.1.1. Consistency Regularization

Consistency regularization enhances model robustness by enforcing prediction invariance under data perturbations or augmentations, improving stability without requiring labeled data. Fu et al. [16] present an SEI method based on dual consistency regularization (DCR) evaluated on a WiFi dataset with 16 categories and an ADS-B dataset with 10 categories. Xu et al. [17] developed an SSL-SEI approach with triple consistency on the 20-class ADS-B dataset.

#### 2.1.2. Generative Modeling

Generative modeling learns latent data distributions rather than class-specific features [18]. Gong et al. [19] integrated auto-encoders with Triple-GAN [20] for signal characterization. Tan et al. [21] developed self-classifying GANs using bispectral features, while Xie [22] and Zhou [23] employed auto-encoder reconstructions for representation learning. However, generative approaches are excluded from our framework due to their computational complexity and tendency to learn features not directly optimized for discrimination. The reconstruction-focused objectives may capture signal characteristics irrelevant to emitter identification, reducing efficiency in practical applications.

#### 2.1.3. Pseudo-Labeling

Pseudo-labeling assigns predicted labels to unlabeled data for model training, bridging semi-supervised and supervised learning. Yang et al. [24] automated labeling with parameter migration to avoid retraining, using a dataset of communication signals from seven radiation sources. Ren et al. [25] employed meta-pseudo-labeling, where a teacher model generates labels for student model training on RF fingerprinting tasks. Pseudo-labeling is avoided for its noise sensitivity, unsuitable for our non-ideal operational recognition with varying signal quality.

### 2.2. Self-Supervised Learning

Self-supervised learning focuses on embedding similarity between augmented samples rather than prediction consistency, learning noise-resistant features without requiring labeled data. Wu et al. [26] combined contrastive learning with pseudo-label selection for AMR, while Wu et al. [27] employed two-stage pretraining for emitter identification. Qiu [28] enhanced classification with UAV and ADS-B datasets. We selected self-supervised learning for its ability to learn robust representations directly from unlabeled signals, effectively handling noise and variations in non-ideal conditions. The SimCLR framework [29] exemplifies this approach by optimizing embedding distances through contrastive learning without label dependencies.

## 3. Problem Formulation

This paper addresses fine-grained radar work mode recognition under non-ideal conditions using semi-supervised learning. We first define fine-grained radar work modes and mathematically model pulse parameter modulation under both ideal and non-ideal conditions, then present our contrastive learning framework.

### 3.1. PDW Multi-Level Hierarchical Model

Radar signals are passively intercepted by ESM receivers, where pulse streams undergo deinterleaving to group pulses from the same emitter [30]. Key parameters, including RF, PW, pulse amplitude, angle-of-arrival, and time-of-arrival, are measured and digitized into PDWs. Statistical parameters derived from PDW sequences, such as PRI, collectively characterize the distinctive features of radar emitters. For MFR, employing diverse signal modes across tasks, we formulate work mode recognition as the pattern recognition of PDW combinations. Using RF, PW, and PRI as core PDW parameters, we define radar work modes and propose a two-level hierarchical representation from an implementation perspective [31].

**Figure 1:**
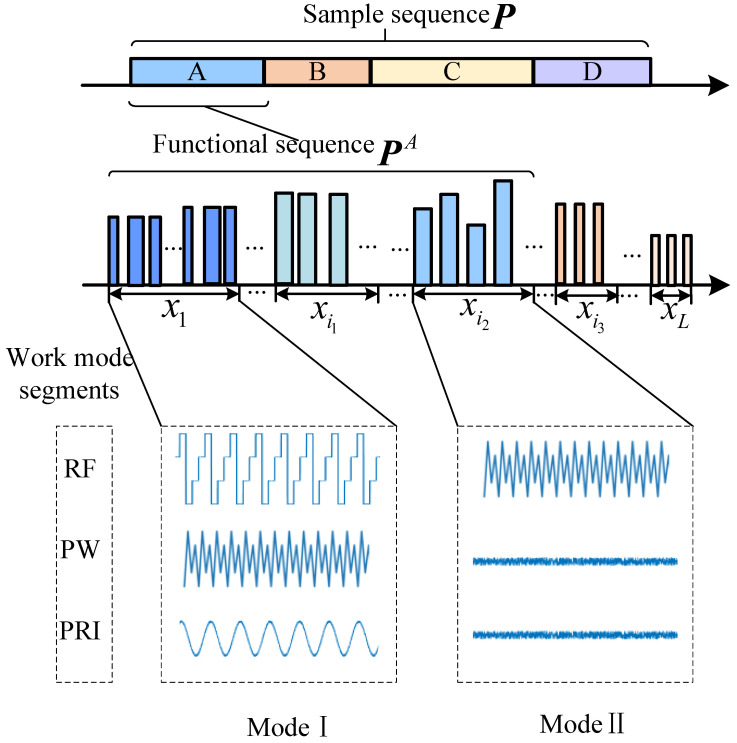
Hierarchical modeling diagram of work modes.

MFR employs a multi-layer signal model to accomplish tasks, relying on a large variety of pulse sequence combinations to achieve higher-level functionalities. This paper models the pulse sequences of MFR from two perspectives: the functional perspective and the modeling perspective, see Figure 1.

From the functional perspective, a radar pulse sequence sample comprises *N* orderedpulses $P=(p_{1},p_{2},\ldots ,p_{N})$. MFR can be configured to schedule diverse pulse sequences for multiple functions, such as search and tracking. $P=\left(P^{A},\ldots ,P^{D},\ldots ,P^{Z}\right)$, where $P^{D}\in R^{P_{M}\times N_{D}}$ is the function D∈[A,Z] within the function sequence, which consists of $N_{D}$ pulses, each pulse characterized by $P_{M}$ parameters. MFR can perform multiple missions by altering the parameter modulation style of the transmitted signal, even in one $P^{D}$. This makes it possible to fulfill various requirements in non-cooperative scenarios, such as tracking, anti-jamming, and improving radar speed and range performance. The subsequences of pulses, composed of parameters with different modulation types, are defined as fine-grained work modes.

From the modeling perspective, a functional sequence sample $P^{D}$, which may encompass multiple work modes, is defined as a sequence of $N_{D}$ consecutive pulses:

$$P^{D}=\left(p_{m_{f}},p_{m_{f}+1},\ldots ,p_{m_{f}+N_{D}-1}\right)\in R^{P_{M}\times N_{D}},$$

where each pulse $p_{t}$ is a vector of $P_{M}$ features (e.g., amplitude, width, and energy), and $m_{f}$ is the starting index of the sequence. This sequence can be partitioned into *L* contiguous segments. A segment $x_{i}$, considered as the minimum identification unit in this paper, consists of $N_{k}$ pulses ($N_{k}\in \left[1,N_{D}\right]$) and serves a specific function and mode:

$$x_{i}=\left(p_{s_{i}},p_{s_{i}+1},\ldots ,p_{s_{i}+N_{k}-1}\right)\in R^{P_{M}\times N_{k}},$$

where $s_{i}$ is the start index of the *i*-th segment. Consequently, the entire sequence sample is described as a collection of these segments: $P=(x_{1},x_{2},\ldots ,x_{L})$. Let $Y=(y_{1},y_{2},\ldots ,y_{L})$ denote the label sequence, where each label $y_{i}$ corresponds to the working pattern of the pulse segment $x_{i}$.

In summary, the MFR constructs signals in a hierarchical manner to optimize and coordinate lower-level pulse sequences for accomplishing mission goals. At the functional level, a pulse sequence sample P consists of *Z* functional sequences. At the operational mode level, each functional sequence is further divided into several mode segments, with the corresponding mode labels stored in the label vector Y.

### 3.2. Modulation Types and Non-Ideal Conditions

Radar systems employ diverse PDW parameter modulations to support multiple functions, including target search, tracking, and missile guidance. We constructed a PDW dataset using three key parameters—RF, PW, and PRI—for radar work mode identification, with modulation patterns determined by mission requirements. RF represents the carrier frequency, enhanced by frequency agility in phased array radars for improved signal processing.PW reflects fundamental radar characteristics, such as short pulses in pulse compression radars.PRI, the interval between pulse arrivals, critically influences range and velocity measurement performance through its inverse relationship with pulse repetition frequency.

For work mode recognition, the modulation modes of PRI and the agility modes of PW and PRI provide critical discriminative information. According to the parameter variation characteristics, the pulse parameter modulation type can be divided into five fundamental forms: constant, jitter, dwell and switch (D-S), stagger, and periodic [14]. Specifically, constant modulation occurs when the parameter fluctuation is less than 1% of the average value; jitter modulation occurs when the fluctuation is more than 30%; D-S modulation is characterized by the parameter value remaining stable in a fixed time period and then jumping; stagger modulation refers to the parameter value around the average value for periodic changes; periodic modulation follows sinusoidal or cosinusoidal patterns.

**Figure 2:**
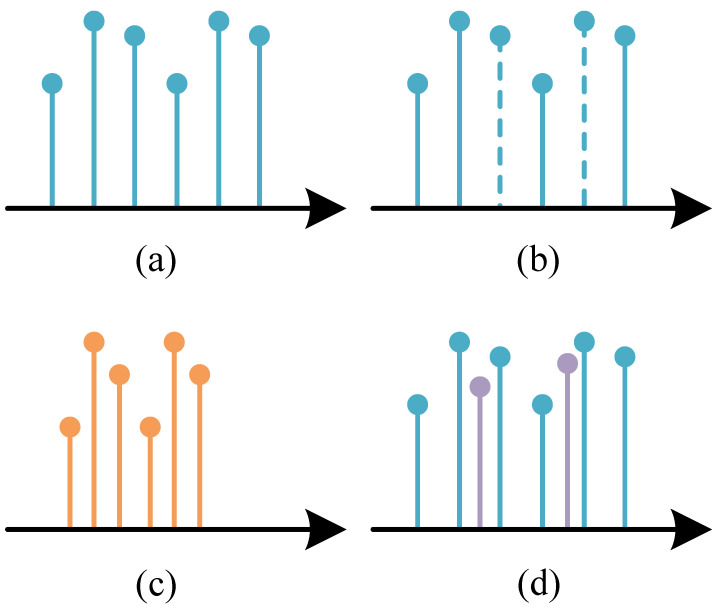
Example of three factors in a non-ideal condition influencing the expected sample of a specific mode. (**a**) Ideal condition. (**b**) Pulse missing. (**c**) Measurement errors. (**d**) False pulse.

Additionally, signals from non-cooperative MFRs are often received under non-ideal conditions, including pulse loss, false pulses, and measurement errors. Figure 2 illustrates the impact of these three degradation factors on radar pulses. Consider the RF parameter within the PDWs as an example: the horizontal axis represents the time sequence, the vertical axis indicates RF, and each vertical line denotes an individual pulse. Figure 2a represents the pulse under ideal conditions. Figure 2b shows the case in which the receiver loses the pulse during the measurement. The receiver fails to receive the corresponding pulse properly due to factors such as long distance and mainlobe misalignment, which results in partial signal loss in the received radar pulse data. Figure 2c shows the distortion of the expected sample in terms of RF and PRI. Figure 2d indicates the insertion of false pulses, where height variations correspond to different RF values and color gradients represent PW variations.

Taking PRI as an example, we mathematically model pulse samples under non-ideal conditions through Equations (1) and (2). Let x denote an original pulse sample with its PRI sequence represented as $pri_{n}$. When *j* consecutive pulses are lost after the *i*th pulse, then the PRI sequence of sample x becomes:

(1) $$x_{pri}=\left\{\begin{array}{cc} pri_{n} & 0<n<i\;\\ \sum_{k=0}^{j}pri_{k} & n=i\;\\ pri_{n+j} & n>i \end{array}\right.$$

When the *k*th pulse in a pulse sample is a false pulse, the total pulse count increases, and the PRI values adjacent to the false pulse are altered.

(2) $$\left\{\begin{array}{c} pri_{k-1}=g_{k}-TOA_{k-1}\;\\ pri_{k}=TOA_{k+1}-g_{k} \end{array}\right.$$

where $g_{k}$ denotes the arrival time of the false pulse.

## 4. Method

**Figure 3:**
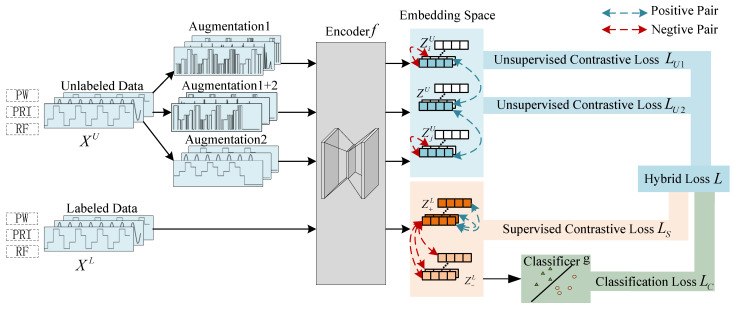
Framework of the proposed approach for the MFR work mode recognition.

This paper proposes TriSCL (Triple-branch Semi-supervised Contrastive Learning), an end-to-end framework for fine-grained radar work mode recognition under non-ideal conditions using limited labeled samples. As shown in Figure 3, the TriSCL model accepts a semi-labeled dataset consisting of unlabeled and labeled samples. Leveraging the signal characteristics of missing and false pulses, we designed two data enhancement functions. Using these functions for augmentation on unlabeled data produces three distinct views: a tail mask view, a timestamp mask view, and a strongly augmented view (combining both techniques). These views are mapped to an embedding space by encoder *f*, where the tail mask view and timestamp mask view try to compute the unsupervised comparison loss by instance with the strongly augmented view, respectively. Meanwhile, the labeled samples go through the encoder to compute the supervised contrast loss in the same embedding space, and then through the classifier to compute the classification loss.

### 4.1. Data Augmentation

To enhance the robustness and generalization capability of radar work mode recognition models under non-ideal conditions, we design two data augmentation techniques for unlabeled samples to simulate pulse loss and false pulse scenarios.

Pulse loss refers to the phenomenon where portions of radar pulses are missing due to noise, interference, or hardware failures. To emulate this condition, we employ a tail masking strategy where the three parameters of the PDW are masked in the posterior part of a sample by a certain percentage. For the input data $X\in R^{B\times T\times C}$, where *B* is the batch size, *T* is the number of pulses in a sample, and *C* is the feature dimension (i.e., the three parameters of the PDW). We calculate the number of pulses to be masked: $L=\left\lfloor T\cdot r\right\rfloor$, where *r* is the masking ratio. The masked data $X_{masked}$ is generated by zeroing the last *L* pulses across all PDW parameters:

(3) $$X_{\mathrm{masked}}\left[b,t,c\right]=\left\{\begin{array}{cc} 0 & \mathrm{if}\;t\geq T-L\\ X[b,t,c] & \mathrm{else} \end{array}\right.$$

False pulses are a non-authentic radar pulse signal that is generated in radar signal processing due to various interferences, noises, or spoofing techniques. To simulate this phenomenon, we adopt a timestamp masking strategy, randomly masking a portion of pulses in a sample pulse train. Specifically, a Bernoulli-distributed mask matrix $M\in {\left\{0,1\right\}}^{B\times T}$ is generated, where each element takes the value 1 (indicating masking) with probability *p*, and 0 (indicating retention) with probability 1−p. This mask matrix is then applied to the input data *X* to obtain the masked data $X_{masked}$.

(4) $$X_{\mathrm{masked}}\left[b,t,c\right]=\left\{\begin{array}{cc} 0 & \mathrm{if}\;M_{b,t}=1\;\\ X[b,t,c] & \mathrm{else} \end{array}\right.$$

**Figure 4:**
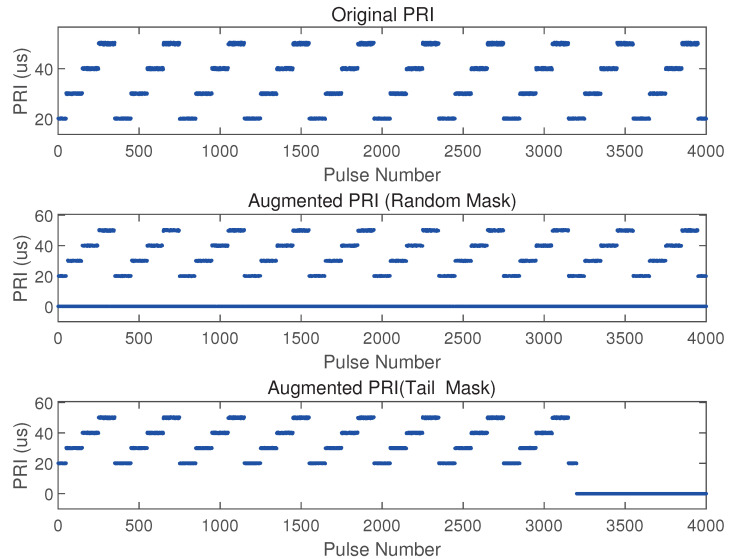
The PRI parameters before and after two types of enhancement.

Figure 4 demonstrates the data before and after applying two data augmentation strategies, using the PRI parameter as an example. Through the two strategies for data augmentation, the model significantly enhances its capability to extract discriminative features, improves adaptability to complex signal conditions, and achieves higher recognition accuracy under pulse distortion scenarios.

### 4.2. Encoder

**Figure 5:**
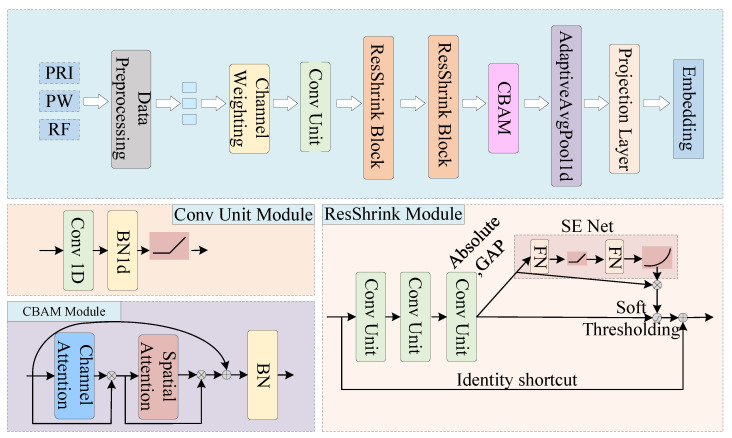
The architecture of the SCARSN encoder.

The encoder will transform input pulse sequences into low-dimensional embedding vectors. These vectors can capture semantic and characteristic information of PDW sequence samples, facilitating subsequent similarity measurement and contrastive loss computation. Different from image processing, signals are usually one-dimensional data. To adapt to the radar pulse dataset, operations such as convolution and pooling used in the encoder are all one-dimensional. Figure 5 shows the structure of the encoder Spatial–Temporal Attention Residual Shrinkage Network (STARSN). The encoder consists mainly of three modules: the convolution unit, the stacked residual shrinkage units, and the spatial–channel attention module. The convolution unit includes a one-dimensional convolutional layer, batch normalization (BN), and the ReLU activation function, which are used to extract the local temporal features of the signal, accelerate convergence speed, and improve computational efficiency. The following provides a detailed introduction to the residual shrinkage module and the spatial–channel attention module adopted in the encoding process.

#### 4.2.1. Spatial and Channel Attention Module

The convolutional operation in neural networks can extract local signal features, but often loses the original temporal information of the signal. While the convolution process treats all channels indiscriminately, each dimension of the PDW parameters contributes differently to radar work mode recognition, necessitating distinct feature extraction strategies. To adaptively emphasize discriminative features while preserving contextual semantic relationships, we firstly introduce the Channel Attention Module (CAM) to guide the encoder network to focus on more discriminative channels and regions. CAM applies global maximum pooling and global average pooling along the feature length dimension of the input feature map $x\in R^{C\times L}$, where *C* denotes the number of feature channels and *L* represents the feature length. This operation compresses the input into two feature vectors of size 1×C. These vectors are then passed through a shared two-layer fully connected (FC) network with input and output dimensions of *C*, where ReLU activation is applied between the layers. The outputs of two branches are combined by element-wise addition, followed by a sigmoid activation function to generate the final channel attention weights $M_{c}$.

(5) $$M_{c}=\sigma \;\left(W_{2}W_{1}f_{\mathrm{avg}}^{c}⊕W_{2}W_{1}f_{\max}^{c}\right)$$

where σ(·) denotes the sigmoid activation function, $W_{1}$ and $W_{2}$ represent the weight vectors of the two fully connected layers, $f_{\mathrm{avg}}^{c}$ and $f_{\max}^{c}$ correspond to the global average pooling and global maximum pooling operations applied over the feature map of size L×C, respectively.

Then, the Spatial Attention Module (SAM) adaptively selects weights of different channels and spatial regions, enabling the network to focus on features with greater influence. The SAM compresses the channel information while maintaining attention to the spatial information of the feature map. First, the global average pooling and global maximum pooling are applied in parallel along the channel dimension to the input feature map $x\in R^{C\times L}$, yielding two feature maps of L×1. These two feature maps are then concatenated along the channel dimension to form a merged feature map of L×2. Finally, a 3×1 convolution operation is employed to fuse the information extracted from both average pooling and maximum pooling, reducing the feature map back to one dimension. The channel attention feature map is obtained after passing through an activation function:

(6) $$M_{s}=\sigma \left({\mathrm{Conv}}_{1D}^{3}\left[f_{avg}^{s},f_{\max}^{s}\right]\right)$$

where $Conv_{1D}^{3}$ denotes a 1D convolution with a kernel size of 3×1, $f_{\mathrm{avg}}^{s}$ and $f_{\max}^{s}$ represent channel-wise global average pooling and global maximum pooling, respectively.

#### 4.2.2. Residual Shrink Module

The primary purpose of introducing this module into the encoder is to improve the ability to extract informative features from noisy samples, eliminate redundant information, and promote classification accuracy. Additionally, by utilizing identity mappings in residual networks, the module facilitates more efficient backpropagation, reduces training difficulty, and mitigates the risk of gradient explosion.

The Residual Shrinkage Module consists of three key components: residual blocks, attention mechanisms, and soft thresholding operations. The residual block is composed of BN, ReLU, and convolution units. These components appear in pairs and also include an identity shortcut connection. Soft thresholding plays a crucial role in many denoising algorithms. Unlike traditional residual networks, the residual shrinkage module embeds an attention subnetwork (SENet), which can adaptively generate thresholds for different samples [32]. Specifically, for a feature map *X* of size L×C, its absolute value is first compressed into a one-dimensional vector of size 1×C through global average pooling. This vector is then fed into an attention network composed of a ReLU activation function and two FC layers to generate a scaling parameter α. Finally, α is mapped to the range [0, 1] by the sigmoid function, as expressed by Equation (7):

(7) $$\alpha =\sigma (W_{2}W_{1}{\left|X\right|}_{avg})$$

where $\left|X\right|$ denotes the absolute value of the feature map and avg represents the global average pooling operation. $W_{1}$ and $W_{2}$ are weight matrices of two FC layers. The threshold τ is obtained by multiplying α with ${\left|X\right|}_{\mathrm{avg}}$.

The soft thresholding operation can identify features that are irrelevant to the current task. It sets the features *x* in the feature map *X* whose absolute values are less than the threshold τ to 0, in order to achieve the goal of denoising. It is implemented using Equation (8):

(8) $$y=\left\{\begin{array}{cc} x-\tau & \mathrm{if}\;x>\tau \\ 0 & \mathrm{if}\;-\tau \leq x\leq \tau \\ x+\tau & \mathrm{if}\;x<-\tau \end{array}\right.$$

where *x* is the input for soft thresholding, and *y* is the output. From Equation (8), it can be seen that the derivative of soft thresholding is either 1 or 0. Therefore, soft thresholding can reduce the risks of gradient vanishing and gradient explosion in deep learning and maintain the stability of gradients during the training process.

### 4.3. Hybrid Loss

For a batch of unlabeled samples $X^{U}$ with batch size *N*:$X^{U}=\left[x_{u1},x_{u2},\ldots ,x_{uN}\right]$, two augmented versions are generated for each sample through distinct data augmentation operations:

(9) $$X_{A}^{U}=\left[x_{Au_{1}},x_{Au_{2}},\ldots ,x_{Au_{N}}\right]$$

and

(10) $$X_{B}^{U}=\left[x_{Bu_{1}},x_{Bu_{2}},\ldots ,x_{Bu_{N}}\right]$$

These augmented samples $X_{A}^{U}$ and $X_{B}^{U}$ will be fed into the feature extraction network and projection network to obtain their embedded representations:

(11) $$Z_{A}=g\left(h\left(X_{A}\right)\right)={\left[{\left(z_{A_{1}}\right)}^{T},\ldots ,{\left(z_{A_{N}}\right)}^{T}\right]}^{T}$$

and

(12) $$Z_{B}=g\left(h\left(X_{B}\right)\right)={\left[{\left(z_{B_{1}}\right)}^{T},\ldots ,{\left(z_{B_{N}}\right)}^{T}\right]}^{T}$$

where $z_{i}\in R^{M}$, *M* denotes the feature dimension. The batch dimensions of $Z_{A}$ and $Z_{B}$ are connected to form $Z\in R^{2N\times M}$. *Z* is used to compute the following contrastive loss function:

(13) $$Z^{\prime }=\left[{\left(z_{A_{1}}\right)}^{T},\ldots ,{\left(z_{A_{N}}\right)}^{T},{\left(z_{B_{1}}\right)}^{T},\ldots ,{\left(z_{B_{N}}\right)}^{T}\right]T$$

Assuming the positive sample feature index for $z_{i}$ is *k*, the loss function to calculate the similarity between the embedding feature of positive and negative samples is denoted as:

(14) $$L=\frac{1}{2N}\sum_{i=1}^{2N}l_{i}$$

(15) $$l_{i}=-\log\frac{\exp(s_{i,k}/\tau )}{\sum_{j=1,j\neq i}^{2N}\exp(s_{i,j}/\tau )}$$

(16) $$s_{i,j}=s\left(z_{i},z_{j}\right)=\frac{z_{i}z_{j}^{T}}{{∥z_{i}∥}_{2}{∥z_{j}∥}_{2}}$$

where $s_{i,j}$ is the cosine similarity between the features of the *i*th and the *j*th sample, τ is the temperature coefficient, and $s_{i,k}$ is the cosine similarity between the features of the *i*th sample and the features of its positive sample.

The loss function, formulated as a combination of the negative logarithm and SoftMax, drives the inner term towards 1. This property can effectively improve the similarity measure between positive sample pairs while reducing the similarity among negative pairs. Given the bounded range of pairwise similarity, the optimization objective of minimizing the loss function can only be reached when the feature distance between positive samples is reduced, and the separation between negative samples is increased simultaneously.

Within the contrast learning framework, the temperature coefficient is mainly used to control the degree of influence of positive and negative sample pairs in the model training process. Taking negative sample pairs as an example, in the high-dimensional feature space, there are significant differences in the similarity performance between different negative sample pairs, with some negative sample pairs presenting higher similarity and others lower similarity. In this case, a smaller temperature coefficient will make the loss function pay more attention to the pairs of negative samples with high similarity. On the contrary, a larger temperature coefficient will help to mitigate over-emphasis on these similar samples, which, in turn, effectively reduces overfitting risks and improves the model’s generalization ability and robustness.

This paper adopts an end-to-end semi-supervised learning framework. Compared with the two-stage contrastive learning method, the proposed method allows the contrastive loss in unsupervised training to directly fine-tune the downstream classifier. At the same time, it can also make full use of the classification loss of labeled data to achieve the unification of model training and optimization, and improve the recognition accuracy for specific tasks [33].

For each unlabeled data $x_{i}^{U}\in X^{U}$, we first employ specialized data augmentation techniques to generate two augmented views and one strongly augmented view that combines both augmentation strategies. The first augmentation applies tail masking with a 30% truncation ratio, processed through encoder *f* to obtain embedding $z_{i}^{U}$, while the second employs timestamp masking via binomial sampling (*p* = 0.2) to produce embedding $z_{j}^{U}$. The strongly augmented view, combining both augmentation strategies, generates a reference embedding $z^{U}$ through the same encoder. Within the contrastive learning framework, embeddings derived from different views of the same sample form positive pairs, while those from different samples constitute negative pairs. We optimize the model using normalized temperature-scaled cross-entropy loss [29] to minimize distances between positive samples and increase the distance between negative samples. The unsupervised loss $L_{U1}\left(f,X^{U}\right)$ is computed between embeddings $z_{i}^{U}$ and $z^{U}$, while $L_{U2}\left(f,X^{U}\right)$ is derived from $z_{j}^{U}$ and $z^{U}$. This dual loss enforces feature consistency across different distortion patterns.

(17) $$L_{U1}\left(f,X^{U}\right)=\frac{1}{N}\sum_{i=1}^{N}-\log\frac{\exp(\mathrm{sim}\left(z_{i}^{U},z^{U}\right)/\tau )}{\sum_{k=1,k\neq i}^{N}\exp(\mathrm{sim}\left(z_{i}^{U},{z_{k}}^{U}\right)/\tau )}$$

(18) $$L_{U2}\left(f,X^{U}\right)=\frac{1}{N}\sum_{j=1}^{N}-\log\frac{\exp(\mathrm{sim}\left(z_{j}^{U},z^{U}\right)/\tau )}{\sum_{k=1,k\neq j}^{N}\exp(\mathrm{sim}\left(z_{j}^{U},{z_{k}}^{U}\right)/\tau )}$$

where sim(·) denotes the similarity function, as defined in Equation (16).

For semi-supervised radar work mode recognition tasks, labeled and unlabeled data may originate from the same dataset with identical distributions. For labeled samples, the shared encoder *f* generates embeddings $z^{L}$ that reside in the same latent space as $z_{i}^{U}$, $z_{j}^{U}$, and $z^{U}$ from unlabeled data. The supervised contrastive loss $L_{s}\left(f,X^{L},Y\right)$ is computed by treating embeddings from the same class as positive pairs and those from different classes as negative pairs. This objective simultaneously maximizes intra-class similarity and minimizes inter-class similarity of the embeddings. For a labeled PDW sample $x_{i}^{L}$ with ground-truth class *y*, its embedding $z^{L}$ is obtained through encoder *f*, and the supervised contrastive loss is formulated as:

(19) $$L_{s}\left(f,X^{L},Y\right)=-\log\frac{\sum_{y=y_{+}}\exp(\mathrm{sim}\left(z_{+}^{L},z^{L}\right)/T)}{\sum_{y=y_{-}}\exp(\mathrm{sim}\left(z_{-}^{L},z^{L}\right)/T)}$$

where $z_{+}^{L}$ denotes the embedding of samples belonging to the same class as $z^{L}$, i.e., $y=y_{+}$, while $z_{-}^{L}$ represents embeddings from different classes, i.e., $y\neq y_{-}$. The optimization of the supervised contrastive loss drives the model to produce more tightly clustered embeddings for intra-class samples while maximizing separation for inter-class instances, ultimately yielding discriminative feature representations that are particularly effective for classification objectives. Subsequently, the labeled sample’s embedding $z^{L}$ is fed into classifier *g* to predict the label $\hat{y}=g\left(z^{L}\right)$. The standard softmax cross-entropy loss $L_{C}\left(f,g,X^{U},Y\right)$ is then computed between $\hat{y}$ and the ground-truth label *y* to optimize the model for radar work mode classification. The classifier comprises a single FC layer with an input dimension of 128 (matching the embedding space) and an output dimension of 6 (corresponding to the number of work mode categories), optimized using cross-entropy loss as the classification objective.

(20) $$L_{c}\left(f,g,X^{U},Y\right)=-\frac{1}{M}\sum_{i=1}^{M}\sum_{c=1}^{C}I(y_{i}=c)\log{\hat{y}}_{c}$$

where ${\hat{y}}_{c}$ represents the predicted probability for class *c* given a labeled sample, while $I(y_{i}=c)$ denotes the indicator function that equals 1 when the true label of the *i*th labeled sample is *c*, and 0 otherwise. In terms of the loss function, the unsupervised contrastive loss learns representations by maximizing the consistency between different augmented views of the same sample. Supervised contrastive learning utilizes the supervised contrastive loss to reduce the distance between samples of the same class in the embedding space. To jointly train this end-to-end framework, the final loss function is a hybrid loss that combines weighted contributions from the unsupervised contrastive loss, supervised contrastive loss, and classification loss.

(21) $$L=\lambda_{1}L_{U1}+\lambda_{2}L_{U2}+\lambda_{3}L_{S}+\lambda_{4}L_{C}$$

where $L_{U1}$ and $L_{U2}$ denote the unsupervised contrastive losses, $L_{S}$ represents the supervised contrastive loss, and $L_{C}$ is the classification loss. The hyperparameters $\lambda_{1}$, $\lambda_{2}$, $\lambda_{3}$, and $\lambda_{4}$ are introduced to balance the contributions of these four loss terms.

During training, the model updates the encoder *f* and classifier *g* by minimizing the hybrid loss, allowing it to learn effective representations from both labeled and unlabeled data while directly optimizing predicted class probabilities to enhance classification performance.

### 4.4. Materials and Datasets

As shown in Table 1, this section conducts simulation experiments using three different datasets, named PDW-N, PDW-MP, and PDW-FP, respectively, where N stands for “Normal”, MP for “Missing Pulse”, and FP for “False Pulse”. Each dataset comprises six categories of fine-grained modes. The PDW-MP and PDW-FP datasets are constructed based on PDW-N to simulate signal quality degradation in practical electronic warfare environments. PDW-MP emulates the missing pulse phenomenon, where pulses are randomly removed from original sequences to replicate conditions caused by receiver insensitivity, electromagnetic interference, or signal occlusion. This configuration evaluates the model’s reasoning capability under incomplete information. In contrast, PDW-FP simulates deceptive false pulse jamming through the injection of noise or interference pulses into clean sequences, representing adversarial electronic countermeasures. This setup assesses anti-jamming robustness and classification stability in noisy and deceptive scenarios. Each pulse is characterized by three modulation parameters, i.e., RF, PW, and PRI. The details of modulation types and parameter ranges considered in this paper are listed in Table 1. Specifically, constant modulation occurs when fluctuation remains below 1% of the average value. Jitter modulation is characterized by fluctuations exceeding 30% of the average value. D-S modulation is manifested as the parameter value remains stable in a fixed time period and then jumps. Stagger modulation refers to the parameter value around the average value for periodic changes. Periodic modulation follows sinusoidal or cosinusoidal patterns.

**Figure 6:**
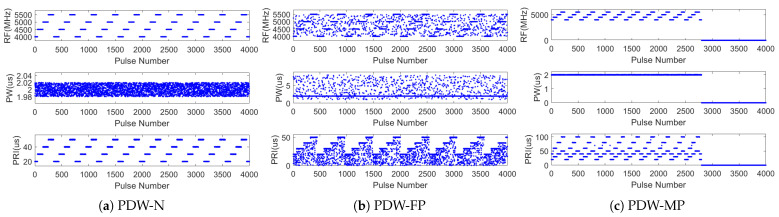
Several examples of three datasets.

Each category of radar work mode in the dataset contains 1000 samples, and each sample is a sequence composed of 4000 pulses. In the PDW-N dataset, the measurement errors of PW and PRI are set to 1%, and the measurement error of RF is ±5 MHz. In the simulation of non-ideal conditions, six scenarios are simulated by using the probability ranges of the occurrence of missed pulses and false pulses, ranging from 0 to 50%, with a step size of 10%. Figure 6 provides some examples of three datasets.

## 5. Experiments and Analysis

### 5.1. Experimental Configuration

Before training, the samples need to be preprocessed to eliminate the influence of the dimensions among different features. The mean value and standard deviation are calculated for all the samples in the training set, validation set, and test set, and the data is transformed into a distribution with a mean of 0 and a standard deviation of 1 by using Equation (22).

(22) $$x_{\mathrm{scaled}}=\frac{x-\mu }{\sigma }$$

The STARSN is composed of adding a residual shrinkage module and a spatial–channel attention mechanism on the basis of ResNet18. Among them, the number of filters in the convolutional layers is 16, 32, and 128, respectively, the size of the convolution kernel is 3×1, and the stride is 1.

The total number of training epochs is set to 100, the batch size is set to 100, temperature coefficient is set to 0.5. We use the AdamW optimizer with an initial learning rate set to 0.001 and a weight decay coefficient of $10^{-5}$. In addition, we introduce a learning rate exponential decay scheduler with warm-up. The warm-up period is 15 epochs, and the exponential decay coefficient is 0.95. In the following experiments, the ratio of labeled to unlabeled data under the default conditions is 1:10. All experimental methods were trained on the PDW-N dataset, where the ratio of the training set to the validation set is 7:3, while evaluated on the PDW-MP and PDW-FP datasets to assess the model’s generalization capability under non-ideal scenarios. The balancing hyperparameters ($\lambda_{1}$, $\lambda_{2}$, $\lambda_{3}$, and $\lambda_{4}$) in the hybrid loss1 (Equation (21)) were manually tuned to ensure comparable magnitudes across all loss terms, guided by established practices in contrastive learning. The final values ($\lambda_{1}$ = 0.3, $\lambda_{2}$ = 0.3, $\lambda_{3}$ = 1.0, $\lambda_{4}$ = 1.0), which achieved optimal performance on the validation set, were adopted in our experiments.

### 5.2. Comparison Experiment

For a comprehensive comparative analysis, the proposed model was evaluated against an extensive set of methods spanning different learning paradigms. The comparison includes representative contrastive learning approaches such as SimCLR [29], BYOL [34], and MOCO [35]. Additionally, classical supervised convolutional networks were considered, including AlexNet [36], VGGNet, ConvNet [37], and ResNet [38]. To ensure a thorough evaluation, the study also incorporates recently developed radar-specific recognition methods, particularly RSCN [12] for noise suppression and BABN [14] for Bayesian uncertainty modeling. To adapt to the radar pulse dataset, all convolutional and pooling operations in these networks were modified to one-dimensional implementations. The supervised methods were trained exclusively using limited labeled samples with cross-entropy loss.

To ensure fair comparison, the training configurations of SimCLR, BYOL, MOCO, RSCN, and BABN were kept consistent with TriSCL. Their data augmentation strategies employed two approaches commonly used for temporal data: scaling augmentation and jitter augmentation. The weak augmentation involved only scaling with a standard deviation factor of 1.1, while the strong augmentation first applied scaling (β=1.1), followed by additive Gaussian noise (β=0.8). All algorithms adopted identical data preprocessing methods.

**Figure 7:**
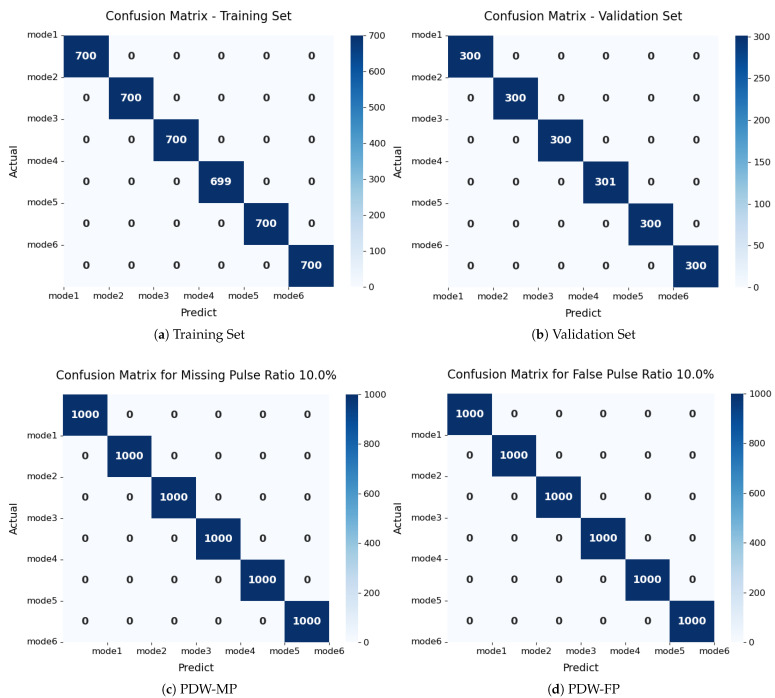
Confusion matrices demonstrating model performance and robustness. (**a**,**b**) show consistent performance between training and validation, indicating no overfitting. (**c**,**d**) demonstrate perfect robustness when handling both PDW-MP and PDW-FP datasets under 10% distortion conditions.

The confusion matrices derived from the training, validation, and challenging test sets (PDW-MP and PDW-FP with 10% distortion) are analyzed to evaluate generalization performance and overfitting, as illustrated in Figure 7. All matrices exhibit a near-diagonal distribution, indicating high classification accuracy. The consistent performance and structural alignment between training and validation sets confirm effective generalization without significant overfitting. Notably, the model achieves 100% accuracy on the test sets containing pulse distortions, demonstrating substantial robustness to interference. This performance is attributed to the integrated framework design: tailored data augmentations emulate realistic signal distortions, while the robust encoder architecture, incorporating residual shrinkage and attention mechanisms, facilitates the extraction of distortion-invariant features. The experimental results collectively validate the generalization capability of the proposed approach and its resilience to targeted signal degradation scenarios.

Table 2 demonstrates the recognition accuracy of these algorithms for multifunctional radar work modes under various non-ideal scenarios. When the proportion of missing pulses is below 20%, the recognition accuracy of our algorithm can reach 100%. As the proportions of missing and false pulses increase, recognition accuracy declines. This occurs because higher distortion proportions compromise discriminative information in the samples and amplify interference and uncertainty during model evaluation. When the proportion of missing pulses is 30%, TriSCL can still maintain a relatively high recognition accuracy for both false pulses and missing pulses, while the accuracies of other algorithms drop significantly. Under high proportions of missing pulses and false pulses, our algorithm still outperforms other supervised and semi-supervised algorithms. Although ResNet shows comparable performance to our method at extremely high interference ratios, our model has a great advantage over ResNet when the proportion is 30% or below. This indicates that the proposed TriSCL can better adapt to the complex electromagnetic environment and enable the model to obtain robust features under non-ideal conditions. At the same time, we found that all models show lower accuracy for missing pulses than for false pulses. This is mainly because missing pulses cause irreversible loss of key signal features, leaving insufficient discriminative patterns for the model to learn; False pulses, while disruptive, still retain authentic signal characteristics that provide learnable features.

**Figure 8:**
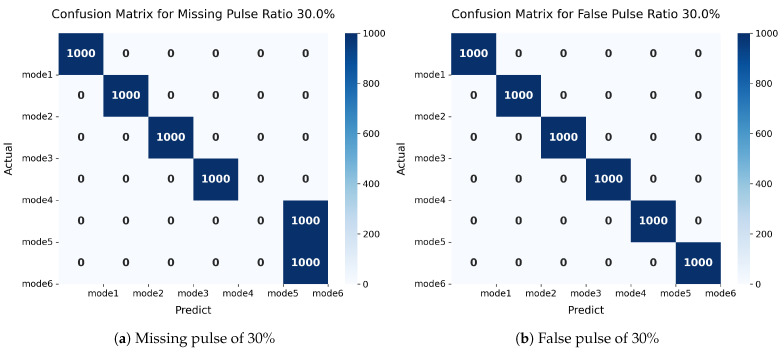
Confusion matrices of TriSCL with the missing ratio of 30% on different datasets.

The confusion matrix analysis demonstrates that the proposed model exhibits confusion between Mode 5 and Mode 6 under 30% pulse missing conditions in Figure 8. As indicated in the parameter configuration Table 1, this misclassification arises from the inherent similarities in their operational parameters: both modes share D-S and Stagger modulation types. Furthermore, 30% pulse missing renders the periodic modulation (in Mode 5) and jitter modulation (in Mode 6) temporally similar in characteristic variations, thereby exacerbating the classification challenge. To mitigate this issue, future work should incorporate a parameter-aware attention mechanism within the feature space, which would adaptively weight the feature representations of different PDW parameters to enhance discriminative capability.

For comprehensive performance assessment, we evaluate our method using three key metrics: classification accuracy, the number of trainable parameters, and Floating Point Operations (FLOPs). All experiments were conducted on the PDW-MP dataset with a 30% missing ratio. It is evident that, compared with other methods in Table 3, the method proposed in this paper has achieved the best performance in terms of performance metrics. While the triple-branch contrastive learning architecture incurs higher computational costs, it also delivers significantly improved accuracy. TriSCL does not have the highest number of parameters or FLOPs; yet, it achieves the highest accuracy, indicating that its model architecture and training strategy are more effective in balancing computational cost and accuracy. Overall, while our method exhibits moderate complexity, it remains feasible with current computing resources and significantly improves performance metrics.

### 5.3. Ablation Study

Table 4 shows the influence of the temperature coefficient on the model’s recognition ability. When the temperature coefficient is 0.6, the model can achieve the best recognition performance, indicating that this parameter value can effectively enhance the model’s adaptability to the environment under non-ideal conditions. Based on this, a temperature coefficient of 0.6 is adopted in subsequent experiments to ensure the model’s robustness.

We analyze the impact of different data augmentation methods within contrastive learning frameworks, comparing our two proposed techniques with common time-series augmentations such as scaling and jitter. As illustrated in Table 5, compared to the proposed methods (tail mask + timestamp mask), the conventional augmentation techniques (scaling + jitter) exhibit inferior overall performance when handling non-ideal scenarios in radar PDW datasets, particularly under high proportions of false and missing pulses. The conventional augmentation methods, although capable of improving the robustness to the value range of PDW features, have significant limitations: they only perform independent perturbations on the value range of pulse features, failing to preserve the inherent temporal correlations and variation trends among pulse sequences. Electromagnetic interference and other noise sources commonly distort pulse sequences during radar receiver operation. The data augmentation methods proposed in this paper simulate these typical non-ideal conditions: Tail Mask simulates the phenomenon of pulse loss, and Timestamp Mask simulates the phenomenon of temporal misalignment. This physics-based augmentation strategy enables the model not only to learn the fault tolerance for feature values but, more importantly, to capture the essential laws of pulse feature variation patterns, thereby maintaining stable recognition performance even under the interference of false pulses and missing pulses.

**Figure 9:**
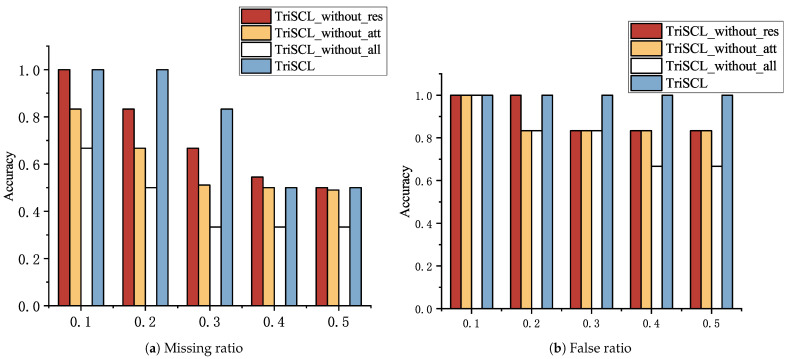
The influence of residual shrinkage and attention mechanism on recognition results.

Next, to validate the impact of the residual shrinkage module and the spatial–channel attention module on the model’s recognition ability, we constructed four comparative models by sequentially removing the attention mechanism, residual shrinkage module, or both. As shown in Figure 9, the recognition performance degrades when these key modules are removed. Moreover, using the residual shrinkage or the spatial–channel attention mechanism module alone can improve the classification accuracy, demonstrating that both the residual shrinkage module and attention mechanism individually contribute to improved classification accuracy. The main reason is that the soft-thresholding operation can adaptively suppress noise, and the spatial–channel attention mechanism emphasizes important feature regions and channels while suppressing irrelevant backgrounds. Experimental results under varying false pulse ratios reveal that the model without the residual shrinkage module and the model without the attention mechanism exhibit comparable performance when the false pulse ratio remains below 20%, with their accuracy declining to 83.33% as the ratio increases. In the recognition results for missing pulses with different proportions, the model without the attention mechanism shows marginally inferior performance compared to the model without the residual shrinkage module. This suggests that the attention mechanism can partially compensate for the absence of the residual shrinkage module. The model with both modules removed demonstrates the poorest performance, confirming the crucial synergistic effect between the residual shrinkage and attention mechanisms. Overall, both modules play vital roles in enhancing the model’s robustness against non-ideal conditions, including varying false pulse ratios and missing pulses.

To evaluate the impact of the number of contrastive learning branches on the recognition performance of the model under various non-ideal conditions, we compared experimental results between two-branch and three-branch configurations. To ensure the validity of the experimental results, except for the loss functions, all other aspects of the three models were kept consistent. The experimental results are shown in Table 6. Among them, the first variant of TriSCL uses only the unsupervised contrastive loss $L_{U1}$, while the second variant employs the unsupervised contrastive loss $L_{U2}$ solely. Both variants, along with the full TriSCL model, exhibit strong performance for false pulses with varying proportions and missed pulses at relatively low proportions, and still hold certain advantages over other semi-supervised methods. When the proportions of false pulses and missed pulses reach 30% or higher, the recognition results of the two variants are relatively close, but both lag behind the three-branch TriSCL model by a certain margin. When the proportion of missed pulses is 30%, the recognition result of TriSCL drops to 0.8333, which is higher than the 0.5 achieved by both variants. Under severe non-ideal conditions, the three-branch architecture demonstrated enhanced stability and recognition capability. This improvement stems from the third branch, which employs a combination of two data augmentations (compared to one in each of the other branches), acting as an extra constraint that generates more contrastive relationships during loss computation. Specifically, it enables dual contrastive calculations between each single-augmentation branch and the composite-augmentation branch, creating multi-layered constraints involving associations between single pulse anomalies (missing or false pulses) and composite pulse anomalies, as well as indirect links between different pulse anomalies through composite scenarios. When calculating contrastive loss, the three branches generate richer contrastive relationships that cover not only individual pulse anomaly patterns but also their combined effects. This prompts the model to learn sample distributions from multiple feature-space dimensions, enabling more generalizable representations that capture the essential patterns amid complex non-ideal conditions. Consequently, performance improves under complex non-ideal conditions—effectively handling false and missing pulses.

To validate the impact of two-stage contrastive learning versus our proposed end-to-end strategy, we conducted comparative experiments on TriSCL using both training paradigms. The two-stage approach (TriSCL-TS) employs triple-branch contrastive learning with dual unsupervised losses ($L_{U1}$ and $L_{U2}$) during pretraining, and transitions to supervised contrastive loss combined with classification loss during fine-tuning. Experimental results in Table 7 demonstrate that the proposed end-to-end TriSCL exhibits excellent robustness, outperforming TriSCL-TS in scenarios with low to medium proportions of false pulses and missed pulses. However, under high proportions of non-ideal conditions, the performance of all models declines; yet, TriSCL remains relatively superior. The key reason for this performance gap is the end-to-end strategy’s superior ability to leverage labeled data. By integrating discriminative feature learning and adaptation to non-ideal scenarios into a unified optimization process, it avoids potential inconsistency between pretraining and fine-tuning stages. This enables the model to maintain stable performance across different proportion ranges of false and missing pulses. In contrast, the performance of TriSCL-TS highlights the limitations of the two-stage training method under the current experimental setup: its separate pretraining (relying solely on unsupervised losses) and fine-tuning stages may lead to a mismatch in feature learning objectives, making it less effective in capturing the complex patterns of pulse anomalies, especially when dealing with varying degrees of non-ideal conditions.

**Figure 10:**
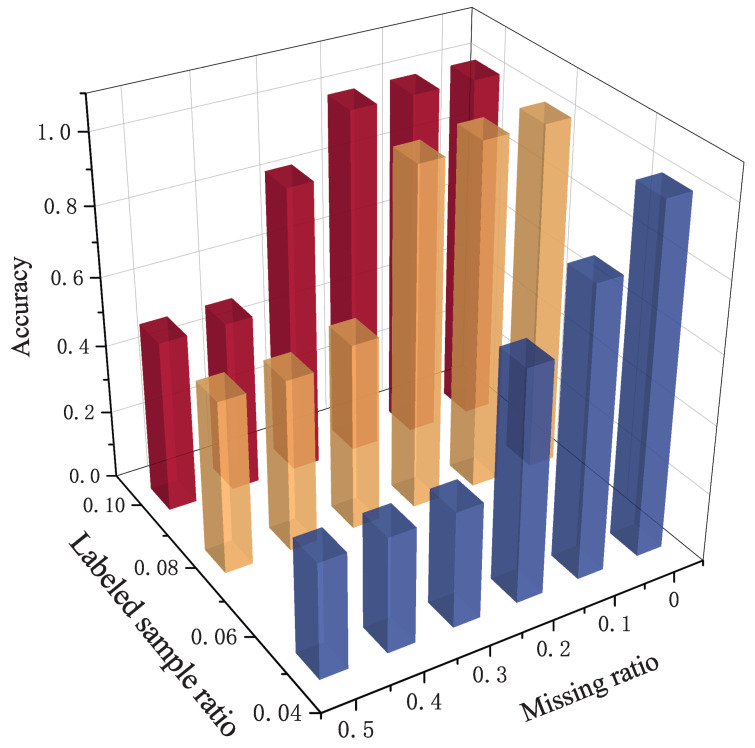
Experimental results under different labeled sample ratios.

As illustrated in Figure 10, recognition performance across all test scenarios significantly improves as the number of labeled samples increases. This occurs because additional labeled data provides the model with richer information about the sample distribution, enabling more discriminative feature extraction. Notably, TriSCL maintains a relatively high recognition accuracy even with extremely limited labeled samples, demonstrating its strong semi-supervised learning capability.

## 6. Conclusions and Future Work

Recognizing fine-grained multifunctional radar modes with limited labeled data is a practically significant, yet challenging task. To address this, we propose a semi-supervised deep learning framework based on triple-branch contrastive learning. This unified framework effectively combines unsupervised contrastive loss, supervised contrastive loss, and classification loss. To bolster robustness against sample quality degradation—specifically false pulses and lost pulses—we introduce tail masking and timestamp masking as novel data augmentation strategies. Furthermore, our encoder incorporates residual shrinkage modules and self-attention mechanisms to adaptively suppress noise and capture key temporal patterns and inter-feature dependencies. Experimental results demonstrate that our method outperforms state-of-the-art supervised and unsupervised approaches, achieving an average accuracy improvement of 15% across varying levels of sample degradation, even when using only 10% labeled data. These results confirm the effectiveness and robustness of the proposed approach.

Future research in radar mode recognition will confront the challenges posed by complex programmable radars, shifting focus to few-shot and zero-shot learning paradigms. This demands models that can generalize to unseen signals by extracting intrinsic features autonomously. Potential paths include metric learning and data augmentation for few-shot learning, graph networks for zero-shot recognition, and hybrid approaches combining sequence matching or envelope analysis with deep models to improve robustness and interpretability. These efforts, though nascent, are crucial for developing systems that operate under authentic battlefield constraints.

## Figures and Tables

**Table 1:** Parameter settings of simulated work modes.

Work Mode	PRI	RF	PW
	20∼50 μs	4000∼5500 MHz	1∼8 μs
MODE1	D-S	D-S	Constant
MODE2	D-S	Stagger	Stagger
MODE3	Constant	D-S	D-S
MODE4	Constant	Stagger	Constant
MODE5	Periodic	D-S	Stagger
MODE6	Jitter	Stagger	D-S

**Table 2:** Recognition results of different DL methods under pulse loss and false pulse conditions.

Compared Methods	ACC (False Pulse (%))						ACC (Missing Pulse (%))					
	0	10	20	30	40	50	0	10	20	30	40	50
AlexNet	100	51	50	50	49.7	33.3	100	50	50	50	48	35.5
ResNet	100	100	100	100	99.9	99.5	100	100	66.7	63	50	49.8
CovNet	100	100	100	100	100	100	100	83.3	66.4	50	50	48
VGG	100	71.4	67.4	50.3	50	50	100	49.7	33.3	33	30	30
SimCLR	100	100	66.7	66.7	60.2	47.1	100	83.3	66.7	66.7	33.3	30
BYOL	100	33.2	16.7	16.1	16.1	16.1	100	16.1	16.1	16.1	16	16
MOCO	100	100	100	83.4	65.1	17	100	66.7	66	50	20	16.7
RSCN	100	100	99.8	83.3	83.3	83.3	100	83.3	66.7	66.7	66.1	31.5
BABN	100	80	32.2	23.7	21.6	20	100	50	31.2	30	17.9	17.1
**TriSCL**	**100**	**100**	**100**	**100**	**100**	**100**	**100**	**100**	**100**	**83.3**	**50**	**50**

*Note*: Bold entries represent the results obtained by the method (TriSCL) proposed in this paper.

**Table 3:** Complexity comparison with other recognition methods.

Methods	Parameters	FLOPs ($\times 10^{6}$)	Accuracy
AlexNet	18,710	4.42	0.50
ResNet	10,390	42.43	0.67
LeNet	3590	10.75	0.50
VGG	12,542	14.11	0.30
SimCLR	60,118	39.28	0.67
BYOL	52,374	42.44	0.17
MOCO	35,606	85.43	0.50
RSCD	19,254	42.44	0.67
BABN	12,396	39.21	0.30
**TriSCL**	**58,189**	**52.62**	**0.83**

*Note*: Bold values correspond to the results of TriSCL, the method proposed in this study.

**Table 4:** Recognition results under different temperature coefficients.

Temperature Coefficient	False Pulse (%)						Missing Pulse (%)					
	0	10	20	30	40	50	0	10	20	30	40	50
0.1	100	100	100	100	99.9	83.3	100	100	96.2	33.3	33.3	33.3
0.2	100	100	100	100	98.1	88.3	100	100	100	33.3	33.3	33.3
0.4	100	100	100	100	100	99.7	100	100	100	70.3	0.5	0.5
0.5	100	100	100	100	100	100	100	100	100	78.3	0.5	0.5
**0.6**	**100**	**100**	**100**	**100**	**100**	**100**	**100**	**100**	**100**	**83.3**	**0.5**	**0.5**
0.8	100	100	100	100	100	100	100	100	99.8	83.3	0.5	0.5
1.0	100	100	100	100	100	100	100	100	97.9	83.3	0.5	0.5

*Note*: The bolded row indicates the temperature coefficient value (0.6) selected in the experiments of this paper.

**Table 5:** Recognition results of different data augmentation methods.

Method	False Pulse (%)						Missing Pulse (%)					
	0	10	20	30	40	50	0	10	20	30	40	50
scale + jitter	100	100	83.3	83.3	83.3	83.3	100	83.3	66.7	66.7	50.0	50.0
**tail mask + timestamp mask**	**100**	**100**	**100**	**100**	**100**	**100**	**100**	**100**	**100**	**83.3**	**50.0**	**50.0**

*Note*: The bolded row represents the results obtained by the data augmentation function proposed and adopted in this paper.

**Table 6:** The influence of the number of branches on recognition results.

Method	False Pulse (%)						Missing Pulse (%)					
	0	10	20	30	40	50	0	10	20	30	40	50
TriSCL-$L_{U1}$	100	100	100	100	100	89.4	100	100	97.4	50	46.1	33.3
TriSCL-$L_{U2}$	100	100	100	100	100	89.5	100	100	97.3	50.0	46.2	33.3
**TriSCL**	**100**	**100**	**100**	**100**	**100**	**100**	**100**	**100**	**100**	**83.3**	**50.0**	**50.0**

*Note*: The bolded row represents the experimental results of the model proposed in this paper (TriSCL).

**Table 7:** The influence of end-to-end training approaches on experimental results.

Method	False Pulse (%)						Missing Pulse (%)					
	0	10	20	30	40	50	0	10	20	30	40	50
TriSCL-TS	16.7	16.7	16.7	16.7	16.7	16.7	16.7	16.7	16.7	11.4	11.4	11.4
**TriSCL**	**100**	**100**	**100**	**100**	**100**	**100**	**100**	**100**	**100**	**83.3**	**50.0**	**50.0**

*Note*: The bolded row represents the experimental results of the model proposed in this paper (TriSCL).

## Data Availability

The data that support the findings of this study are available from the corresponding author upon reasonable request.

## Abbreviations

The following abbreviations are used in this manuscript:

EW	Electronic Warfare
ES	Electronic Support
EA	Electronic Attack
EP	Electronic Protection
MFR	Multifunction Radar
RF	Radio Frequency
PW	Pulse Width
PRI	Pulse Repetition Interval
LSTM	Long Short-Term Memory
CNN	Convolutional Neural Network
TriSCL	Tri-branch Semi-supervised Contrastive Learning
SEI	Specific Emitter Identification
DCR	Dual Consistency Regularization
SSL	Semi-Supervised Learning
ADS-B	Automatic Dependent Surveillance–Broadcast
GAN	Generative Adversarial Network
SimCLR	A Simple Framework for Contrastive Learning of Visual Representations
AMR	Automatic Modulation Recognition
UAV	Unmanned Aerial Vehicle
ESM	Electronic Support Measures
D-S	Dwell and Switch
STARSN	Spatial-Temporal Attention Residual Shrinkage Network
BN	Batch Normalization
ReLU	Rectified Linear Unit
CAM	Channel Attention Module
FC	Fully Connected
SAM	Spatial Attention Module
SENet	Squeeze-and-Excitation Network
